# Efficacy of Bee Venom Acupuncture for Chronic Low Back Pain: A Randomized, Double-Blinded, Sham-Controlled Trial

**DOI:** 10.3390/toxins9110361

**Published:** 2017-11-07

**Authors:** Byung-Kwan Seo, Kyungsun Han, Ojin Kwon, Dae-Jean Jo, Jun-Hwan Lee

**Affiliations:** 1 Department of Acupuncture & Moxibustion, Kyung Hee University Hospital at Gangdong, 892, Dongnam-ro, Gangdong-gu, Seoul 05278, Korea; seohbk@hanmail.net; 2 Clinical Research Division, Korea Institute of Oriental Medicine, Daejeon 34054, Korea; hks8158@kiom.re.kr (K.H.); cheda1334@kiom.re.kr (O.K.); 3 Department of Neurosurgery, Kyung Hee University Hospital at Gangdong, 892, Dongnam-ro, Gangdong-gu, Seoul 05278, Korea; apuzzo@hanmail.net; 4 Korean Medicine Life Science, University of Science & Technology (UST), Campus of Korea Institute of Oriental Medicine, Daejeon 34113, Korea

**Keywords:** bee venom acupuncture, pharmacopuncture, low back pain, chronic pain

## Abstract

Bee venom acupuncture (BVA) is an effective treatment for chronic low back pain (CLBP) through the pharmacological effects of bee venom and the simultaneous stimulation of acupoints. However, evidence of its efficacy and safety in humans remains unclear. Using a double-blind, randomized study, 54 patients with non-specific CLBP were assigned to the BVA and sham groups. All participants underwent six sessions of real or sham BVA for 3 weeks, in addition to administration of 180 mg of loxonin per day. The primary outcome, that is, “bothersomeness” derived from back pain, was assessed using the visual analog scale. Secondary outcomes included pain intensity, dysfunction related to back pain (Oswestry Disability Index), quality of life (EuroQol 5-Dimension), and depressive mood (Beck’s depression inventory). Outcomes were evaluated every week during the treatment period and followed up at weeks 4, 8, and 12. After 3 weeks of the treatment, significant improvements were observed in the bothersomeness, pain intensity, and functional status in the BVA group compared with the sham group. Although minimal adverse events were observed in both groups, subsequent recovery was achieved without treatment. Consequently, our results suggest that it can be used along with conventional pharmacological therapies for the treatment of CLBP.

## 1. Introduction

Chronic low back pain (CLBP) is one of the most common, expensive, and disabling musculoskeletal conditions that last longer than 7 to 12 weeks, and it is characterized by frequent recurrence but without underlying pathological causes [1]. Research suggests that the chronic progression of low back pain is associated with demographic factors, disease-related conditions (multiple functional symptoms, pain in the legs, significant disability at onset, multiple recurrences or in-hospital treatment), occupation, underlying spinal condition, and psychosocial state [2]. The socioeconomic burden of CLBP is associated with various comorbidities, increased prescriptions of pain-relieving pharmacotherapy, and increased healthcare service utilization [3]. Research also suggests that CLBP patients with associated symptoms, including depression, anxiety, and insomnia, tend to obtain healthcare services more frequently. Among them, 36% of the patients received combination therapy [3]. A cohort study demonstrated that more than 51% of patients with LBP received complementary and alternative medical (CAM) therapy during their one-year follow-up [4]. Among the alternative therapies, acupuncture is considered a possible option for the treatment of CLBP [5,6]. A systematic review also proposed that acupuncture may have a favorable effect on CLBP [7]. 

Pharmacopuncture is a new acupuncture technique that involves injecting herbal medicine into acupoints, thus combining the therapeutic effect of herbal medicine with that of acupuncture. Bee venom acupuncture (BVA) is a representative pharmacopuncture therapy that originated from bee sting therapy. It is also known that BVA could exert a better analgesic effect than needle acupuncture [8]. Previous research suggests that BVA ameliorates pain by exerting the pharmacological effect derived from bee venom with the simultaneous stimulation of acupoints [9,10]. A study investigating the use of pharmacopuncture in musculoskeletal patients from a Korean medicine hospital reported that 98.6% of inpatients received pharmacopuncture treatment; among them 32.1% received BVA [11]. On the basis of the clinical experiences with the pain-relieving properties of BVA, it is most frequently administered for experimental and clinical studies involving musculoskeletal diseases [12]. As an acupuncture-related modality, BVA has been used to relieve pain by injecting purified and diluted bee venom into acupoints [10] 

Although the pain-relief property of BVA has been reported in animal experiments [9,13,14] and clinical recommendations [10,15,16], there is a scarcity of evidence from rigorous clinical trials [17]. A clinical trial comparing the use of BVA and sham BVA for the treatment of chronic back pain showed that 4 weeks of BVA treatment leads to a decrease in the intensity of pain [17]. However, clinical evidence based on substantial therapy is still lacking. As a matter of fact, to maximize the therapeutic effect of BVA, a gradual increase in the dose is essential. Clinically, dose increment protocol has been widely used for various reasons. By increasing the injection volume or by decreasing the dilution rate, the amount of bee venom injected into each acupoint can be increased. Adaptation to low concentrations of bee venom can minimize side effects and by gradually increasing the dose, the therapeutic effect can be maximized at the same time. Moreover, most of the patients with CLBP prefer combination therapy instead of BVA treatment alone. In this context, the current study was designed to investigate whether the combined treatment of dose increment BVA and non-steroidal anti-inflammatory drugs (NSAIDs) could effectively treat CLBP. 

## 2. Results

### 2.1. Study Participants

**Figure 1:**
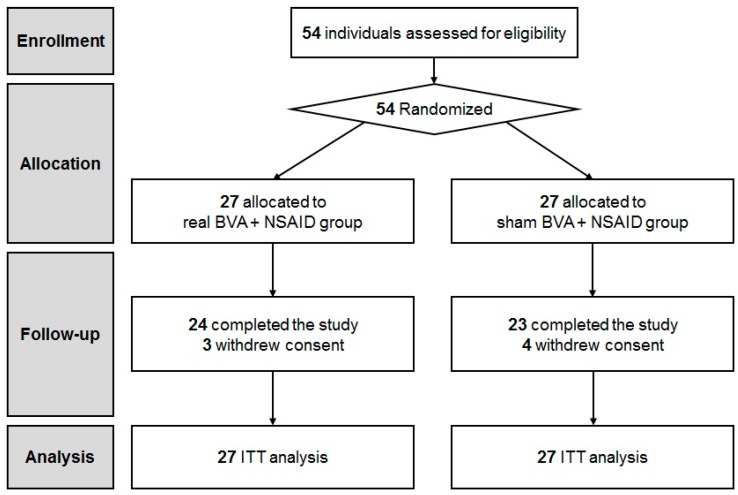
CONSORT flow diagram. BVA: bee venom acupuncture; NSAIDs: non-steroidal anti-inflammatory drugs.

We screened 54 patients and assessed them for eligibility. Ultimately, 54 CLBP patients were enrolled into the study. (Figure 1). Participants were randomly allocated to either the real (*n* = 27) or sham BVA group (*n* = 27). No significant differences were observed in the baseline characteristics and clinical featuresbetween the two groups (Table 1). A total of 24 participants in the BVA group and 23 participants in the sham BVA group completed all the treatment sessions and follow-up evaluations. Three subjects from the BVA group and four subjects from the sham BVA group withdrew their consent. 

### 2.2. Primary Outcome

**Figure 2:**
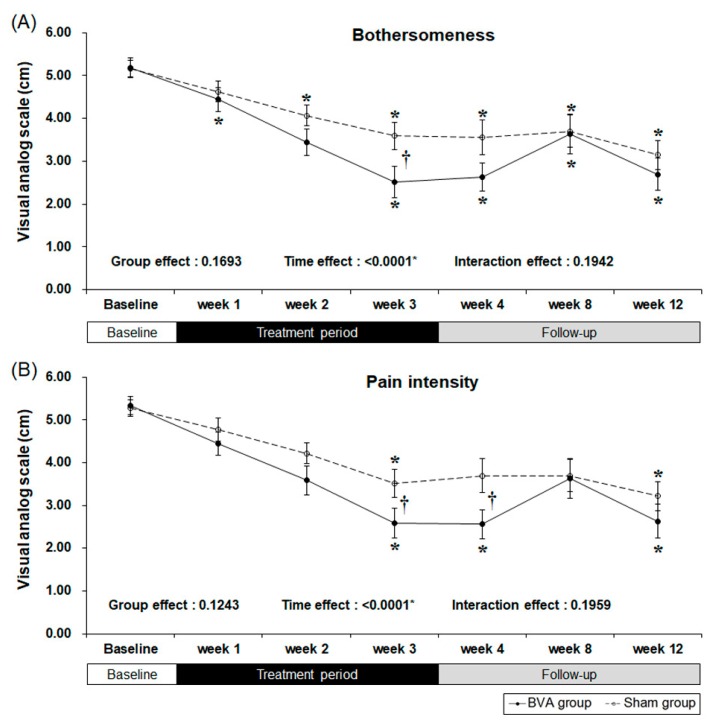
Reduced symptoms as a result from chronic back pain after 3 weeks of bee venom acupuncture (BVA) or sham treatment. (**A**) Changes in visual analog scale (VAS) scores for bothersomeness (**B**) Changes in VAS scores for pain intensity. * Significant difference of pre-post comparison within groups. ^†^ Significant difference between two groups at each time point (*p* < 0.05).

Our primary outcome measure was “bothersomeness,” measured by the visual analog scale (VAS) [18]. Both the BVA and sham groups showed a significant decrease in bothersomeness (Figure 2 and Table 2). Our results showed that three weeks of BVA treatment was superior to sham treatment with an observed least squares mean differences of −1.09 [95% CI −1.98, −0.21] (*p* = 0.0164). As there was no significance in interaction effect (*p* = 0.1942), this can be interpreted that group effect and time effect independently affected the result. Group effect, which is differences between groups due to subjects’ individual differences did not affect the “bothersomeness” measures (*p* = 0.1693). However, time effect, including treatment period and follow-up period, was a significant factor affecting the outcome measure (*p* <0.0001). Decreased bothersomeness persisted after treatment in both the groups; however, there was no difference between the two groups. 

### 2.3. Secondary Outcomes

Secondary results were the pain intensity, disability, quality of life, and depression as measured by the VAS, Oswestry Disability Index (ODI), EuroQol 5-Dimension (EQ-5D), and Beck’s Depression Inventory (BDI), respectively. After three weeks of BVA or sham treatment, pain intensity and back pain-related dysfunction decreased significantly in both groups (Table 2 and Figure 2). However, the subjects treated with BVA showed greater improvements in pain intensity and back pain-related dysfunction than the subjects in the sham group. At our primary endpoint, which was after three weeks of intervention, pain intensity, represented by the VAS score, significantly decreased in the BVA group when compared to that in the sham group (least squares mean difference of −0.95 [95% CI −1.89, −0.01], *p* = 0.0486) and the difference persisted for a week. 

**Figure 3:**
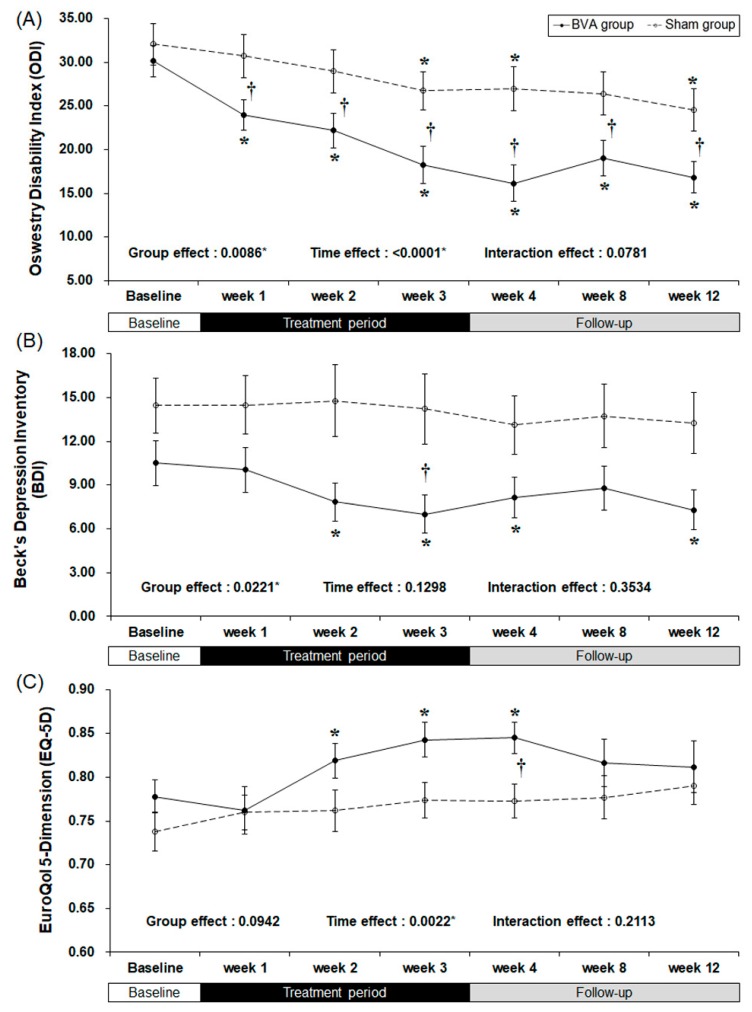
Bee venom acupuncture (BVA) improved the functional and psychological status related to chronic back pain. (**A**) Changes in back pain related dysfunction. (**B**) Changes in back pain related depressive symptoms (**C**) Changes in back pain related quality of life. * Significant difference of pre-post comparison within groups. ^†^ Significant difference between two groups at each time point (*p* < 0.05).

There were clinically meaningful changes in the ODI score (Table 2 and Figure 3). At baseline, there were no significant differences between the groups. As the study treatment continued, the ODI score started to improve significantly during the first week of the trial (least squares mean difference of −7.67 [95% CI −13.29, −2.05], *p* = 0.0085), although in the BVA group after the first week it decreased continuously throughout the 12 weeks of study. The difference between the groups maximized (10.81 points) at week 4.

The BDI score also decreased significantly in the BVA group during weeks 2, 3, and 4 (*p* = 0.0026, 0.0002, and 0.0162, respectively), but no significant difference was observed in the sham group throughout the study. The difference between the two groups was the greatest (least squares mean difference of −4.86 [95% CI −9.70, −0.02], *p* = 0.0492) by the end of the treatment period (week 3). However, group effect, which is the difference between two groups from the baseline affected the result. 

The overall quality of life of both groups improved as shown by an increase in the EQ-5D scores. However, only the real BVA group showed significant improvement at weeks 2, 3, and 4 (*p* = 0.0029, 0.0009, and 0.0005, respectively), while the only the EQ-5D score of the sham group changed significantly at week 4 (least squares mean difference of 0.056 [95% CI 0.000, 0.103], *p* = 0.0278), which was one week after the completion of treatment.

### 2.4. Credibility Analysis

Both the BVA and sham groups showed high credibility in all four categories of the credibility test (Table 3). There was no significant difference among the groups. Moreover, no significant change was observed within both groups after the treatment period.

### 2.5. Adverse Events

Six patients from the BVA group (22.2%) and four patients from the sham group (14.8%) reported adverse events. Four patients from the real BVA group experienced a minimal itching sensation, but recovered completely without any treatment. Two patients from the real BVA group and four patients from the sham group experienced non-specific complaints, including headache (two in sham BVA and one in real BVA), generalized myalgia (one in real BVA), and dizziness (two in sham BVA). There was no significant difference among the groups (*p* = 0.484). 

## 3. Discussion

To the best of our knowledge, this is the first randomized controlled trial (RCT) that has compared increasing doses of BVA and sham treatment as an adjunct to NSAIDs for CLBP patients. Our results showed that three weeks of pragmatic BVA treatments using the protocol for dose increments could be effective for the relief of bothersomeness, pain intensity, functional status related to CLBP, and quality of life. These superior effects were observed during the treatment period and seemed to last during the nine weeks of follow-up, without any harmful reactions. BVA exerts its pharmacological actions through the bioactive compounds isolated from bee venom [15]. 

Despite the clinical favorability and broad utilization for pain management [12], evidence from rigorous RCTs to investigate the effectiveness of BVA in CLBP is limited [10]. A prospective observational study reported that CAM treatment with BVA had a better effect on pain relief and the ODI score than conventional treatment in patients with lumbar intervertebral disc herniation [19]. There were few RCTs regarding the efficacy of BVA for low back pain. Kim et al. reported that five sessions of BVA improved low back pain when compared with sham BVA (normal saline injection) [20]. In a randomized, sham-controlled, triple-blind, two-group parallel clinical trial, Shin et al. [17] demonstrated that eight sessions of BVA at six acupoints (0.1 mL BVA for each acupoint) over 4 weeks improved pain intensity, function, and the quality of life; however, at the time of the eighth treatment the pain intensity was not significantly different between the two groups. 

We attempted to reproduce the actual clinical environment by using an adjunctive BVA with NSAIDs protocol and incremental bee venom protocol. Along with rigorous blinding measures among the participants, assessor, practitioner, and statistician, our credibility analysis showed that participants in both the groups expected a logical treatment and believed that both treatments were effective. To minimize the non-specific and placebo effects the participants from both groups were treated using the same protocol. Based on previous studies, clinical experience, and through a consensus of the experts, all treatment procedures, including the BVA protocol and acupoints, were rigorously designed so that they could not be discriminated.

Our study yielded surprising results in the recovery of the overall symptoms of CLBP. As opposed to the results from the sham group, the primary outcome (bothersomeness) significantly decreased after 3 weeks (six sessions) of BVA treatment. VAS scores for pain intensity showed an analogical tendency. The follow-up observation after treatment revealed that the decreased bothersomeness and pain lasted for a week. However, 5 weeks after the treatment was completed, the symptoms were not statistically different between the groups. Nonetheless, in both groups, the improvement in symptoms from baseline persisted. 

There are several probable reasons why both groups showed significant improvement. First, all the subjects in our study took an analgesic during the treatment period. On observation during the follow-up (without any treatment), we found that the therapeutic effect of the BVA treatment persisted and was superior to that in the sham group. Second, the sham BVA treatment may have stimulated acupoints when the normal saline was injected, possibly yielding an effect similar to that of acupuncture. 

A similar clinical study with BVA (0.1 mL BVA for each acupoint) and sham BVA for back pain showed a significant decrease in the pain intensity in the two groups, although no significant differences were observed between the groups [17]. This corresponds to our study results that sham BVA can also contribute to pain relief. However, our study is unique as we used incremental doses of BVA, instead of using a single dose of bee venom throughout the study. We gradually increased the injection volume every week. This implies that the incremental doses of BVA were the dominant factor for the overall improvement observed in our study. 

Studies have revealed that the analgesic effect of BVA is partially mediated by the descending pain inhibitory system, including the activation of the α2-adrenergic receptor [21], as well as the modulation of immune responses including the reduction of c-Fos expression [22]. The anti-inflammatory activity of BVA is related to the inhibition of COX-2 activity and the production of pro-inflammatory cytokines (TNF-α and IL-1β) [23], as well as the induction of apoptosis through the activation of caspase-3 [24]. 

Other than the analgesic effect of BVA, we also found several other noteworthy results. BVA treatment not only reduced the intensity of pain but also decreased the degree of disability related to CLBP. “Bothersomeness” was developed as a symptom-based health outcome measure for chronic disease [25] and has been used as a severity measure in primary care, with suitability for patients with LBP as well as physicians [18,26,27]. The bothersomeness of low back pain measures the severity of symptoms correlated with pain and disability among primary care patients with nonspecific low back pain and can be a predictor of outcome [18]. Bothersomeness is a measure of pain, functional status, work absence, psychologic health, and other LBP factors [18,28,29]. Its reliability and suitability is validated in LBP studies [30]. The decreased VAS for bothersomeness in our study indicates improvement in the perception of clinical severity and its impact on daily life of patients with CLBP. Surprisingly, unlike the previous study of Shin et al. [17], outstanding improvement was observed in the ODI score. A significant reduction in back pain-related dysfunction, as assessed by the ODI score, persisted throughout the 12 weeks of follow-up. Quality of life as assessed by EQ-5D was significantly reduced by the BVA from the treatment sessions until the 1-week follow-up. Furthermore, depressive symptoms, as assessed by BDI, were significantly reduced only in the BVA group, but not in the sham group. 

A systematic review regarding bee venom (BV) demonstrates that adverse events related to BV are frequent and practitioners should be cautious with its concentration, especially when increasing the dose [31]. The BV-specific responses, such as pain, swelling, redness, and itching of skin, could be detected by patients and doctors. A study with 130 cases of BVA treatment in a Korean medicine hospital reported that 28.5% of patients suffered from an itching sensation [32]. A meta-analysis comparing BVA and normal saline showed a 3.61-times as high a risk of adverse events in BVA [31]. Studies emphasized that a skin test for hypersensitivity reaction is needed to prevent adverse events. Our clinical study demonstrated several minor adverse events, which were less than we expected. Despite the incremental dose protocol, skin tests performed prior to the study prevented the incidence of critical adverse events. 

Our study has a few limitations which should be taken into consideration when interpreting the results. Although we successfully blinded the BVA and sham treatment groups, incorporating another sham group with a non-invasive treatment may have demonstrated pronounced results. Moreover, to find the maximum tolerable injection dose in humans, groups with different concentrations of BVA should be considered in future experiments.

In conclusion, our results have established clinical evidence that BVA could improve bothersomeness, pain intensity, functional status, and the quality of life of patients with CLBP. This suggests that BVA treatment may be a potential candidate along with conventional pharmacological therapies for the treatment of CLBP.

## 4. Materials and Methods

### 4.1. Study Design

This was a balanced-randomized, double-blinded, placebo-controlled, parallel-group study conducted at the Kyung Hee University Hospital at Gangdong (KHUHGD) from April 2011 to August 2013. We enrolled patients with CLBP to receive either one of the following two regimens: six sessions of BVA with NSAIDs or sham injection with NSAIDs over three weeks. This study was conducted following the rules of the Declaration of Helsinki. According to the delegation of responsibilities log, each study step, such as treatment preparation, acupuncture practice, outcome measurement, study coordination, data management, and statistical analyses, were performed by independent researchers. The study was approved by the Institutional Review Board (IRB) of KHUHGD (approval number: KHNMC-OH-IRB 2011-006), and registered at ClinicalTrials.gov (number NCT01491321). A full detail of the trial protocol was disclosed in a previous publication [33].

### 4.2. Sample Size Calculation

The sample size was estimated based on a prior acupuncture study on CLBP that used a 10 cm VAS pain score as a measure of the primary effectiveness. The mean difference and common standard deviation of 10 cm in the VAS pain score (mean difference = 1.5, SD = 2.73) were used to determine the sample size [34]. Assuming a two-sample *t*-test model for two-armed study, 27 patients per group were necessary with power to be 80%, level of significance 5% (two-sided), and an anticipated dropout rate of 20%. Interim analysis was not considered in this study.

### 4.3. Eligibility Criteria

Patients were recruited through advertisements in local newspapers, on hospital websites, and bulletin boards. Eligibility was determined using the inclusion and exclusion criteria (Table 4) [33]. Written informed consent was obtained from the participants after receiving an explanation of the study protocol. Participants were educated about the possible adverse events, including itching, local swelling, erythematous change, and a hypersensitivity reaction. The participants were also informed that they could stop their participation at any stage without any disadvantages. The participants were excluded from the study if they refused to continue, withdrew their consent, violated enrollment criteria or trial protocol, or participated in less than four treatment sessions. Participants were also informed that the trial could be stopped in case of unacceptable risks of serious adverse events or severe clinical deterioration by the principal investigator; they would be treated and compensated based on the reimbursement agreement. 

This study included both men and women in the age group of 18 to 65 years with non-specific, uncomplicated LBP for at least three months, scoring ≥4 points on a 10 cm VAS for bothersomeness from LBP, and without any abnormalities on neurological examination (e.g., lumbosacral nerve function, deep tendon reflexes, plantar response, voluntary muscle activation, and sensory function). As a part of the screening procedure, a skin hypersensitivity test was performed on all the patients [33]. The exclusion criteria of the study were as follows: subjects with radicular pain, serious spinal disorders, chronic diseases that could affect or interfere with the therapeutic outcomes, previous spinal surgery, painful conditions induced by traffic accidents, apparent musculoskeletal pain other than back pain, conditions such as clotting disorders, administration of an anticoagulant agent, pregnancy and seizure disorders where BVA might not be safe; patients with documented hypersensitive reactions to previous BVA treatments, bee stings or insect bites; severe psychiatric or psychological disorders; patients who were using corticosteroids, narcotics, muscle relaxants or herbal medicines to treat low back pain, or any medications considered inappropriate by the investigator at the time of the study were excluded from the study. Furthermore, subjects with pending lawsuits/receipt of compensation due to LBP, those who refused to participate in the trial or provide consent and unable to read and write in the Korean language, were excluded from the study.

### 4.4. Randomization and Blinding

Participants were randomly allocated to different groups. The randomization sequence was generated using the Statistical Package for the Social Sciences (SPSS, Inc., Chicago, IL, USA) by an independent statistician. The enrolled researchers, practitioner, and outcome assessors were blinded to the random allocation sequence using sequentially numbered, sealed, opaque envelopes until the trial completion. The randomization and allocation concealment process was supervised by the principal investigator. 

To keep the study practitioner blinded to the allocation, pharmacopuncture (either real or sham BVA) was prepared by an independent treatment facilitator and transferred to the practitioner. The practitioner was further blinded to the patients’ allocation and was only allowed to discuss about the disease. The outcome assessors remained blinded to the patients’ treatment allocation until the end of the trial. Case Report Form (CRF) data entry was conducted by independent researchers and was thoroughly cross-checked for integrity and accuracy. Statistical analysis was performed by an independent statistician who was blinded to the allocation information. 

### 4.5. Interventions

Before the pharmacopuncture treatment, a skin hypersensitivity test was performed on all patients at the LI11 acupoint by subcutaneously injecting 0.05 mL of a 20,000× bee venom preparation. The patients were excluded from the study if they presented with local swelling that exceeded over 10 mm (in diameter) or redness over 20 mm (in diameter).

Pharmacopuncture was conducted by a certified acupuncturist (licensed Korean medical doctors (KMD)) having more than 10 years of clinical experience. The participants received a total of six pharmacopuncture sessions over three weeks using either real or sham BVA, respectively. BVA was prepared with dried bee venom powder (Yoomil Garden, Hwasun, Korea) diluted to 20,000× in normal saline (0.9% NaCl) and filtered. The same amount of normal saline was prepared as the sham BVA. Both diluted bee venom and normal saline appeared identical to the bare eyes when they were prepared in syringes. Participants were treated while in the prone position after sterilization of their skin. Both the real BVA and sham BVA treatments were subcutaneously injected following a pre-defined weekly incremental protocol [33]. Injection volume for each acupoint was gradually increased from 0.2 mL for the first week, 0.4 mL for the second week, and 0.8 mL for the third week. The dose increment protocol was decided by a consensus of specialists of Korean medicine. Generally, first injection starts with 0.1–0.2 mL. The injection volume is increased every session or every other session by 0.1–0.2 mL until reaching the maximum volume of 0.8–1.2 mL [32]. Pharmacopuncture was performed perpendicularly at a depth of 0.5–1.0 cm at 10 acupoints (Shenshu [BL23], Qihaishu [BL24], Dachangshu [BL25], Huantiao [GB30], Yaoyangguan [GV3], Mingmen [GV4], Xuanshu [GV5]).

The most widely used NSAID, loxonin (Loxoprofen, 60 mg/Tab, Dongwha Co., LTD., Seoul, Republic of Korea) was prescribed to both the BVA and sham groups (dosage: three tablets per day for three weeks). A conventional medical doctor prescribed the oral medication to both the groups and independent pharmacists dispensed the loxonin, counted, and disposed the returned drugs based on the Institutional Review Board (IRB) guidelines. All the participants underwent a self-administration exercise education program for the management of CLBP.

### 4.6. Outcomes Measures

Baseline demographic characteristics, such as age, gender, medical history, smoking, and drinking were collected using a questionnaire. Independent researchers who were blinded to the group allocation and were not involved in intervention conducted a series of measurements for the bothersomeness of CLBP, pain intensity, back pain-related dysfunction, impact on quality of life, and depressive symptoms at the 1st, 2nd, 3rd, 4th, 8th, and 12th weeks of the trial. The primary endpoint of this study was the time point at which subjects completed six sessions of either BVA or sham treatment, which was the third week of the intervention. We examined the credibility of a sham pharmacopuncture at the baseline and at the end of the treatment, while adverse events were monitored at every visit.

As a primary outcome, the participants were asked to report their degree of bothersomeness from CLBP within the past week at every visit, using a 10 cm VAS (0, absence of bothersomeness; 10, the worst bothersomeness imaginable) [29]. 

Pain intensity within the past week was evaluated using 10 cm VAS [35,36]. To evaluate back pain-related dysfunction, a validated Korean version of the ODI was used [37,38]. The ODI consists of 10 categories of daily activities, including inventories of pain intensity, personal care, lifting, walking, sitting, standing, sleeping, sexual life, social life, and traveling. Each of the response categories was given a score from 0 to 5. The Korean version of the EQ-5D [39,40] consisted of five questions regarding mobility, personal care, daily activities, pain/discomfort, and anxiety/depression rated on a scale of 1 to 3 that evaluated the impact on the quality of life of the patients with CLBP. The Korean version of the BDI [41,42] consisted of 21 questions regarding depressive symptoms and was scored on a scale of 0 to 3. 

The validated Korean version of the credibility test [43] consisted of four questions regarding the expectation of improvement for CLBP, willingness to recommend to others, rationality of treatment, expectation of improvement for other diseases, and was used to assess the discrimination of real-sham treatment allocation, rated on a scale of 1 to 6.

### 4.7. Statistical Analysis

The data were collected and managed by an independent researcher, as described previously [33]. Intention-to-Treat (ITT) analysis was used using the last observation carried forward (LOCF) method. Analysis of covariance (ANCOVA) was performed for the outcome variables and a paired *t*-test or Wilcoxon signed rank test was used to compare results within groups. Using repeated-measures analysis of variance (RM ANOVA), interactions between time and treatment were explored. A chi-square or Fisher’s exact test was used for qualitative data. Statistical analyses were performed with SPSS statistical software package version 18.0 (SPSS Inc., Chicago, IL, USA). A *p* value less than 0.05 was considered statistically significant.

## Figures and Tables

**Table 1:** Baseline characteristics of the participants from BVA and sham groups.

Characteristics	BVA Group (*n* = 27)	Sham Group (*n* = 27)	*p*-Value
Gender (Male/Female) ^†^	9 (33.33%)/18 (66.67%)	4 (14.81%)/23 (85.19%)	0.1115
Age (years) ^‡^	49.85 (14.44)	50.07 (11.06)	0.7175
Height (cm) ^§^	162.03 (7.65)	160.35 (7.15)	0.4099
Weight (kg) ^§^	64.09 (11.02)	61.60 (10.26)	0.3928
Vital sign			
SBP (mmHg) ^§^	122.78 (12.63)	120.26 (15.34)	0.5131
DBP (mmHg) ^§^	75.56 (8.10)	72.93 (10.48)	0.3071
Pulse (times/minute) ^‡^	75.81 (9.85)	78.44 (10.30)	0.3763
Temp (°C) ^‡^	36.15 (0.28)	36.10 (0.26)	0.4681
Smoke (Yes/No) ^††^	3 (12.00%)/22 (88.00%)	0 (0.00%)/27 (100.00%)	0.1041
Drink (Yes/No) ^†^	10 (40.00%)/15 (60.00%)	6 (23.08%)/20 (76.92%)	0.1929
VAS for bothersomeness (mm) ^‡^	5.19 (1.14)	5.16 (1.07)	0.9073
VAS for pain (mm) ^‡^	5.33 (1.11)	5.28 (1.02)	0.8745

BVA: bee venom acupuncture; SBP: systolic blood pressure; DBP: diastolic blood pressure; VAS: visual analog scale. Data shown as mean (standard deviation). ^†^ Chi-square test, ^‡^ Wilcoxon rank sum test, ^§^ Independent *t*-test, ^††^ Fisher’s exact test.

**Table 2:** Comparison of treatment and follow-up response after 3 weeks of bee venom acupuncture intervention.

Outcome	Time	BVA Group (*n* = 27)		Sham Group (*n* = 27)		Within (BG) ^†^	Within (PG) ^†^	*p*-Value ^‡^
		Mean	SD	Mean	SD			
VAS score								
Bothersomeness								
	Baseline	5.19	1.14	5.16	1.07			
	week 3	2.52	1.89	3.59	1.67	<0.0001 *	<0.0001 *	0.0164 *
	week 4	2.63	1.74	3.56	2.08	<0.0001 *	<0.0001 *	0.0548
	week 8	3.63	2.42	3.70	1.96	0.0061 *	0.0002 *	0.8823
	week 12	2.70	2.00	3.15	1.77	<0.0001 *	<0.0001 *	0.3502
Pain intensity								
	Baseline	5.33	1.11	5.28	1.02			
	week 3	2.59	1.82	3.52	1.70	<0.0001 *	<0.0001 *	0.0486 *
	week 4	2.56	1.80	3.70	2.03	<0.0001 *	0.0005 *	0.0273
	week 8	3.63	2.42	3.70	1.96	0.0083 *	0.0002 *	0.9069
	week 12	2.63	2.06	3.22	1.76	<0.0001 *	<0.0001 *	0.2478
ODI score								
	Baseline	30.14	9.17	32.07	12.48			
	week 3	18.25	11.16	26.76	11.28	<0.0001 *	0.0100 *	0.0085 *
	week 4	16.15	10.71	26.96	13.01	<0.0001 *	0.0259 *	0.0018 *
	week 8	19.06	10.60	26.40	12.84	<0.0001 *	0.0955	0.0349 *
	week 12	16.81	9.34	24.54	12.51	<0.0001 *	0.0407 *	0.0171 *
BDI score								
	Baseline	10.52	7.99	14.44	9.66			
	week 3	7.00	6.72	14.22	12.59	0.0026 *	0.0719	0.0429 *
	week 4	8.15	7.23	13.11	10.32	0.0002 *	0.0677	0.1670
	week 8	8.78	7.76	13.74	11.37	0.1963	0.1337	0.2525
	week 12	7.30	7.12	13.26	10.78	0.0064 *	0.2018	0.0765
EQ-5D score								
	Baseline	0.778	0.097	0.738	0.115			
	week 3	0.843	0.102	0.774	0.106	0.0009*	0.1567	0.0511
	week 4	0.845	0.095	0.773	0.100	0.0005*	0.1793	0.0278 *
	week 8	0.816	0.141	0.777	0.128	0.0997	0.1978	0.5776
	week 12	0.812	0.155	0.790	0.110	0.1317	0.0961	0.9381

BVA: bee venom acupuncture; VAS: Visual Analog Scale; ODI: Oswestry Disability Index; BDI: Beck’s Depression Inventory; EQ-5D: EuroQol 5-Dimension; ^†^ *p*-value by Wilcoxon signed rank test or paired *t*-test (ODI and BDI of BVA group and EQ-5D of total group); ^‡^ *p*-value by Analysis of covariance (ANCOVA), adjusting for baseline scores; * *p* < 0.05.

**Table 3:** Comparisons of treatment credibility before and after the treatment period.

Outcome	Time	BVA Group (*n* = 27)		Sham Group (*n* = 27)		Within (BG) ^†^	Within (PG) ^†^	*p*-Value ^‡^
		Mean	SD	Mean	SD			
Credibility test								
Improvement expected								
	Baseline	5.15	0.60	4.89	0.58			
	week 3	5.15	0.66	4.85	0.66	0.9999	0.9999	0.3211
Recommendation to others								
	Baseline	4.67	0.78	4.56	1.01			
	week 3	4.74	0.66	4.67	0.83	0.9999	0.8359	0.8491
Treatment logical								
	Baseline	4.89	0.80	4.41	0.84			
	week 3	4.89	0.58	4.67	0.78	0.9999	0.1826	0.9938
Effective also for other diseases								
	Baseline	4.48	1.09	4.56	0.93			
	week 3	4.70	0.82	4.52	1.16	0.2500	0.9648	0.2349

^†^ *p*-Value by Wilcoxon signed rank test; ^‡^ *p*-Value by Analysis of covariance (ANCOVA) adjusting for baseline scores.

**Table 4:** Inclusion and exclusion criteria of the study.

**Inclusion Criteria**	
Age between 18 to 65 years old	
Experienced low back pain for the previous three months or more.	
Scoring more than 4 points on a 10 cm Visual Analog Scale (VAS) for bothersomeness of low back pain	
Exhibiting no abnormalities on neurological examination (for example, lumbosacral nerve function, deep tendon reflexes, plantar response, voluntary muscle activation, and sensory function)	
Having non-specific, uncomplicated low back pain that qualifies as the following International Classification of Diseases 10 codes:	
M513	Other specified intervertebral disc degeneration
M545	Low back pain
M548	Other dorsalgia
M549	Dorsalgia, unspecified
S335	Sprain and strain of lumbar spine
S336	Sprain and strain of sacroiliac joint
S337	Sprain and strain of other and unspecified parts of the lumbar spine and pelvis
Participants who agreed and signed the informed consent	
**Exclusion criteria**	
Back pain with radicular pain	
Serious spinal disorders, including malignancy, vertebral fracture, spinal infection, and inflammatory spondylitis	
Other chronic diseases that could affect or interfere with the therapeutic outcomes, including cardiovascular disease, diabetic neuropathy, active hepatitis, fibromyalgia, rheumatoid arthritis, dementia, and epilepsy	
History of spinal surgery or subjects scheduled for spinal surgery during the study	
Pain induced by traffic accidents	
Musculoskeletal pain other than back pain	
Conditions that can be aggravated by bee venom treatment, including: clotting disorders, administration of anticoagulant agents, pregnancy, and seizure disorders	
Hypersensitive reactions to previous bee venom treatments, bee strings or insect bites	
Severe psychiatric or psychological disorders	
Current use of corticosteroids, muscle relaxants, narcotics, or herbal medicines. Use of any medication considered inappropriate by the investigator	
Pending lawsuit or receipt of compensation because of low back pain	
Subjects who refused to participate in the trial or provide informed consent	
Subjects unable to read and write in Korean language	
